# Assessing the Reliability of Compliance with the General Treatment Recommendations by Patients Treated for Temporomandibular Disorders

**DOI:** 10.3390/jcm14186674

**Published:** 2025-09-22

**Authors:** Małgorzata Pihut, Wojciech Maga, Andrzej Gala

**Affiliations:** Dental Institute, Prosthodontic and Orthodontic Department, Medical College, Jagiellonian University, 4 Montelupich Str., 31-155 Krakow, Poland; malgorzata.pihut@uj.edu.pl (M.P.); andrzej.gala@uj.edu.pl (A.G.)

**Keywords:** TMD, management of TMD, therapeutic recommendations, muscles relaxation exercise, physiotherapy, glucosamine, chondroitin, sleep hygiene

## Abstract

**Background/Objectives**: The aim of this study was to assess the accuracy of TMD patients’ adherence to treatment recommendations, given in writing, based on an anonymous survey. **Methods:** The study material included a group of 80 patients of both sexes, aged 21 to 45 years, who came for prosthetic treatment due to symptoms of TMD at the Department of Prosthetics and Orthodontics, Jagiellonian University Medical College in Krakow. Axis I of the DC/TMD was used in the diagnosis of dysfunction. The study used an anonymous questionnaire survey, which asked specific questions regarding the reliability of the implementation of the therapeutic recommendations contained in the written treatment instructions, given to patients at the first diagnostic visit. The questionnaire survey was completed by patients once, at the second visit, which was routinely made after 4 weeks. **Results:** The analysis showed that the most frequent adherence of respondents was to physiotherapy treatments. The same number of patients (57.5% each) used sleep hygiene, stress management, and maintenance of dental arch dislocation during the day. More than half of the subjects used orthopaedic pillows during sleep and performed daily relaxation exercises. Less than half of the subjects (46.3–47.5%) used hot compresses on the masticatory muscles, took prescribed supplements, controlled the position of the jaw, and used an occlusal splint at the required time. **Conclusions:** The results of the study indicate a low percentage of adherence to the recommendations made by the doctor.

## 1. Introduction

Temporomandibular disorders (TMD) are now a very common disorder that affects people in their third and fourth decades of life in the stomatognathic system, with a higher frequency in women. At the same time, the age of patients with the painful form of this disease is decreasing, which is a serious problem in the development of adolescents. It is the name for a syndrome of changes involving morphological and functional imbalances within the symmetrically functioning temporomandibular joints under physiological conditions, abnormalities of the work of the muscles of the anterior and middle facial parts of the skull, and the mutual spatial relationship of the dental arches [1,2,3,4,5]. It is now believed that one of the main etiological factors responsible for the development of TMD is excessive stress, lack of coping skills, and unawareness of its effects on the stomatognathic system [6,7,8,9].

The main symptoms of parafunction are wedge-shaped defects in the area of the tooth necks, damage to the tooth enamel, maceration of the buccal mucosa in the occlusal line, impressions on the tongue, and pathological wear of natural teeth and prosthetic restorations. Often, these intraoral symptoms indicate long-term occurrence of occlusal and non-occlusal parafunctions and are most often unconscious.

Among the many methods of treating TMD, the use of occlusal splints, physiotherapy treatments, patient exercises, and the “fight” against occlusal parafunctions, or habits, which are responsible for a very high load on the musculoskeletal system, are important. Every patient should be informed of the highly harmful effects of parafunctional activities. They have a detrimental effect not only on the masticatory system but also on the muscles of the neck, shoulder girdle, and hearing. The temporomandibular joints and the masticatory muscles are most at risk. Pathomorphological overload of soft tissue structures occurs in these joints, leading to serious damage. Tenderness and thickening of the muscles develop, indicating persistent, excessive strain. Many ultrasound examinations of temporomandibular joints confirm that long-term excessive joint loading generates numerous changes, such as damage to the posterior ligament of the joint disc, hypertrophy of the synovial membrane, retrograde sclerotic changes of the joint surfaces, and a heterogeneous image of the joint disc.

It should be carefully explained to the patient that the independent use of occlusal splints or physiotherapy alone will absolutely not bring good and lasting results in the treatment of TMD [10].

A serious mistake in the treatment of TMD is to omit a thorough explanation to patients about the impact of stressors and the inability to cope with stress on the development of this condition. At the same time is a failure to provide comprehensive information regarding the causes of the disorder’s development and to present treatment recommendations verbally and in a cursory manner. This has adverse consequences for all therapy. The latter aspect is very important in therapy, so each patient should receive treatment recommendations in writing so that at home they can reread them and analyze what was communicated at the visit. Often, patients who report prosthetic treatment require an occlusal splint. This results in the treatment process being overlaid with therapy, which primarily involves addressing occlusal and non-occlusal parafunctions. Understanding the need to completely stop clenching or grinding your teeth is essential for achieving good and lasting results in TMD treatment [1,3,6]. Patients must be made aware that psychosocial stressors and inadequate coping strategies are key etiological factors in the development and perpetuation of TMD. The importance of written instructions is emphasized, as they allow patients to revisit and better understand the therapeutic advice at home.

Therapeutic recommendations for the patient are a very important section of self-therapy and therapy, which is indispensable in the treatment of this condition. Since only the patient decides what position the mandible is currently in, and it is the patient who must abandon occlusal parafunctions (grinding, clenching teeth, or tapping them), only the patient himself can “train” to abandon this very harmful habit [2,5,11,12,13,14,15]. This is one of the most important aspects of successful and long-lasting TMD treatment. During therapy, the patient must keep mental control, what is happening to his or her jaw position during the day and understand that dental arch dislocation is very important and necessary for the health of the mouth [1,4,9].

Conscientious adherence to therapeutic recommendations is a very important aspect, determining the success of treatment. It should be noted that this issue is very rarely addressed in the literature, and much more attention should be paid by physicians treating TMD patients to the need to verify patients’ compliance with recommendations [1,6,8,14]. Therefore, we highlight the role of physicians in actively verifying and monitoring compliance at subsequent visits.

At the same time, it is very important for the patient to follow other recommendations, regarding the use of supplements containing glucosamine and chondroitin (ingredients beneficial for the soft tissue elements of the temporomandibular joints), as well as the use of an orthopedic pillow, and performing daily muscle exercises aimed at relaxing the mastication muscles. Patient education regarding important aspects of treatment also includes emphasizing the importance of behavior in the afternoon and evening hours and avoiding excessive emotions, intense physical exercise, thoughts about difficult issues, and stimulating beverages (coffee, energy drinks). Another important piece of information for the patient is the need to maintain a distance of approximately 3–4 mm between the dental arches.

Taking into account the main etiological factor, which is psychoemotional disorders, it is important for the patient to work on his or her emotions, because agitation and neuroticism contribute to a large extent to the development of TMD. Therapeutic recommendations provided to the patient should include information on finding methods of mental relaxation, educating on how to cope with stress, and better organizing daily duties.

The aim of this study was to assess the accuracy of TMD patients’ adherence to treatment recommendations, given in writing, based on an anonymous survey.

## 2. Materials and Methods

The study material included a group of 80 patients of both sexes, aged 21 to 45 years, who came for prosthetic treatment due to symptoms of TMD (pain of the masticatory muscles, limited opening of the mouth, popping and clicking in the temporomandibular joints) at the Department of Prosthetics and Orthodontics, Institute of Dentistry, Jagiellonian University Medical College in Krakow. Patients were recruited for the study from January 2024 to May 2025. Axis I of the DC/TMD (International Network for Orofacial Pain and Related Disorders Methodology; 2018) [2,16] was used in the diagnosis of dysfunction. The first group of patients consisted of patients with the painful form of temporomandibular disorders (29 women and 11 men), and the second group (II) consisted of patients with the painless form of the dysfunction (30 women and 9 men). Both groups included patients diagnosed.

With both stage I a and II a according to DC/TMD. The inclusion criteria for the study were a diagnosis of I a and II a according to DC/TMD, good general health, an appropriate age range of patients, and consent to participate in the study. The exclusion criteria were progression of TMD to a more advanced form (I b, c, II b, III a), the desire to withdraw from the study, and the occurrence of general diseases that prevented participation in the study.

The study used an anonymous questionnaire survey (in our own development), which asked specific questions regarding the reliability of the implementation of the therapeutic recommendations contained in the written treatment instructions given to patients at the first diagnostic visit. The recommendations were explained and given to the patient in written form for each individual patient. During this conversation, the necessity of following all recommendations was also justified, due to the patients’ required self-therapy.

The questionnaire survey included the following questions: whether the patient used: daily performance of 15 repetitions of relaxation exercises throughout the day, hot compresses for the masticatory muscles, taking the indicated supplements (glucosamine and chondroitin), combating stress/education of ways to cope with stress, use of physiotherapy treatments in a series of 10–14 treatments, use of an orthopedic pillow, sleep hygiene, mental control of jaw position/fighting the pathological habit of clenching teeth during the day, required duration of use of an occlusal splint. The questionnaire survey was completed by patients once, at the second visit, which was routinely made after 4 weeks.

Statistical calculations were performed using IBM SPSS Statistics 29.0. An analysis of basic descriptive statistics was performed, along with the Shapiro-Wilk test for the recommendation adherence rate index. This index was created by summing up the recommendations followed—the higher the score, the more recommendations were followed. In order to compare patient groups (pain group vs. non-pain group), an analysis was performed using Student’s *t*-test for independent samples. To determine the relationship between group membership and adherence to individual recommendations, a χ^2^ (chi-square) test analysis of independence was performed. Analogous analyses were performed for the relationship between gender and adherence to individual recommendations. α = 0.05 was used as the level of significance [17].

To determine differences in adherence to recommendations by gender and group membership, a two-factor analysis of variance was performed in a 2 × 2 scheme.

## 3. The Results

Table 1 presents an analysis of the frequency of adherence to each recommendation. The analysis showed that the most frequent adherence of respondents was to physiotherapy treatments in a series of 10–14 treatments in small intervals. The same number of patients (57.5% each) used sleep hygiene, stress management, and maintenance of dental arch dislocation during the day. More than half of the subjects used orthopaedic pillows during sleep and performed daily relaxation exercises. Less than half of the subjects (46.3–47.5%) used hot compresses on the masticatory muscles, took prescribed supplements, controlled the position of the jaw, and used an occlusal splint at the required time.

**Figure 1:**
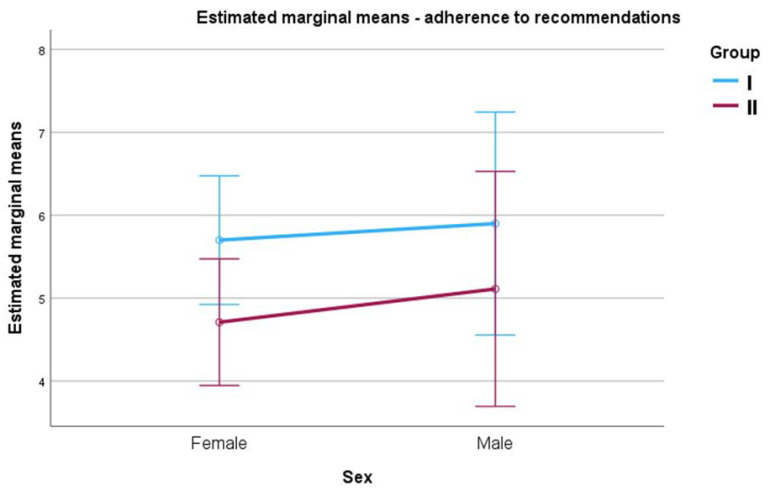
Estimated marginal averages for adherence by group membership and gender of patients.

For the created index of the degree of adherence to recommendations, a descriptive statistics measure was calculated on the basis (Table 2). The possible range of scores was from 0 to 10. The average score was 5.28, which means that the surveyed patients, on average, adhered to about 5 recommendations. Analysis by Shapiro-Wilk attestation showed that the distribution was skewed from a normal distribution, with visual assessment of the distribution (Figure 1) and analysis of the skewness values allowing us to assume that the results assumed a distribution close to a normal distribution.

### Adherence Rate vs. Presence of Pain Symptoms and Gender of Subjects

To determine whether the presence of pain symptoms and gender as factors differentiate the degree of adherence, a two-factor analysis of variance was conducted in a 2 × 2 scheme. The analysis did not confirm the presence of differences between groups, F (1.76) = 2.51; *p* = 0.117; η^2^ = 0.03, and between men and women, F (1.76) = 0.29; *p* = 0.594; η^2^ < 0.01. The interaction of the two factors was also found to be non-significant, F (1.76) = 0.03; *p* = 0.858; η^2^ < 0.01. This result indicates similar adherence rates between groups, between men and women, and when both factors were considered simultaneously. The estimated marginal means are illustrated in Figure 1.

## 4. Discussion

The goals of treatment for TMD include the reduction of pain and improvement of jaw function. Most of the articles [1,3,6,9,18,19] talking about the general principles of treatment describe methods such as the use of occlusal splints, intramuscular injections of botulinum toxin, intraarticular injections of hyaluronic acid, arthrocentesis, and principles of pharmacological and psychological treatment. In the available literature, the authors have not found works that emphatically emphasize the role of implementing general therapeutic recommendations to be followed by patients, regardless of the form of TMD. At the same time, information was not available in the analyzed articles. The key issue is the need to eliminate the occlusal parafunctions, such as clenching or grinding teeth, which is why patients are often unaware of these mechanisms. When the doctor asks about teeth clenching, the frequent response is that they do not clench their teeth during the day, perhaps at night. The literature frequently reports that these parafunctions occur simultaneously.

The authors’ own clinical experience over many years indicates a low level of compliance with treatment recommendations by patients treated for TMD. This is related to the low level of knowledge of patients about the causes of TMD and the principles of treatment, which is why the conversation and explanations provided by the doctor during the first visit are often not sufficient. This limited knowledge contributes to insufficient initial education, which requires reinforcement during treatment.

Patients themselves, during subsequent physical examinations, admit that they do not have time to do the prescribed exercises, have not found an appointment for physiotherapy, do not apply hot compresses to the masticatory muscles, and have not yet addressed better stress management. Importantly, we have in mind general recommendations that apply to most patients in terms of the type of TMD. It should be emphasized that performing exercises on your own, e.g., every 1.5 h, is not only intended to relax the muscles, but also to remind the patients of the necessary exclusion of the dental arches [1,2,5,7,20,21,22]. We acknowledge that such practical barriers, including limited time, difficulty scheduling physiotherapy, and inadequate stress management, represent significant obstacles to adherence.

One of the most important reasons for patients’ compliance with non-surgical treatment recommendations is the need to relieve pressure on the temporomandibular joint structures and create conditions for healing of the soft-tissue elements of the joints (the posterior disc ligaments) by maintaining the discus of both dental arches and relaxation exercises for the masticatory muscles. This also ensures a rapid decrease in musculoskeletal pain [15,17]. In this context, compliance is essential to reduce joint overload and to promote healing of the soft-tissue structures, particularly the posterior disc ligaments.

Wright E. and Klasser G [1] in their handbook emphasize the importance of this patient activity, due to the large spectrum of benefits in the “fighting” against TMD. The authors emphasize the complexity of TMD and the extremely important principle of treatment, which is the need to implement several directions of therapeutic procedures simultaneously. This fact is rarely understood by patients.

Martins W et al. [21] indicate in their publication that manual musculoskeletal methods are effective in the treatment of TMD. In the short term, the latter has a greater effect than other conservative treatments for TMD. In turn, Doeuk C et al.’s [22] low-level laser treatment (what the authors suggest in their recommendations for physiotherapy) is currently being used for various disorders, with focus on its applications in wound healing, scarring, and disorders of the TMD. In this condition, close attention should also be paid to co-occurring diseases unrelated to the masticatory system.

Wänman A. et al. [6] list the treatment methods as grouped as follows: behavior treatment, jaw exercises, sensory stimulation, pharmacological therapy, occlusal appliances, occlusal correction, and temporomandibular joint surgery. Evaluation of treatment effect was based on patient-important outcomes that included pain intensity, physical functioning, emotional functioning, and global rating of improvement, but no mention was made of the importance of reviewing general recommendations, extremely important for overall first-line intervention treatment. The same authors stress the importance of exercise for body posture and jaws. The aim of posture exercise and coordination training is to increase patients’ body awareness and reduce loads that negatively affect joints and muscles. The aim of passive stretching is to improve mobility—the length of the muscle and the range of movement of the TMJ; stretching may also help patients overcome feelings of fear to move the jaw. Seven randomized and controlled trials (RCTs) involving 304 patients with primarily myofascial pain and subjected to posture exercise were identified. The studies examined the effect of posture training, activation against resistance, or both, compared to counseling.

The results of the conducted studies indicate unsatisfactory compliance with the recommendations, which may constitute an implication for the promotion of knowledge in this area and the education of primary care physicians on preventive factors in this regard. This is an inspiration to disseminate knowledge about the need to follow therapeutic recommendations by patients and their verification by physicians involved in the treatment of TMD. Due to the many etiological factors of TMD, the treatment plan for each patient must be considered individually, but general principles are very helpful in managing this group of patients. The order of the steps is also important, as failure to follow this rule can lead to serious errors and a lack of positive treatment outcomes. We additionally emphasize that raising awareness among primary care physicians about prevention and adherence is of high importance.

De Freitas et al. [14] conducted an analysis of counseling and providing important information to patients and found that counselling- and self-management-based therapies could be considered as important, low-cost, and beneficial treatment alternatives for treating TMD to potentially improve psychological domains and remove harmful behaviors for the control of the signs and symptoms of TMD. However, it should be noted that patients are often very unhappy with the fact that they have to admit that they have not followed the given recommendations and become rude to the doctor who conducts the check-up. In such a situation, it is necessary to explain again the significant importance of the patient’s own work in achieving a satisfactory result of TMD treatment and the importance of his participation in this complex process.

Li et al. [23] indicate conservative treatment methods, which include the use of patient education, behavioral, physiotherapy, physical therapy, laser, pharmacologic, and occlusal appliance splint therapies. Unfortunately, when broadly describing TMD treatment methods, the authors do not pay attention to the importance of self-therapy and following therapeutic recommendations. Patient participation in TMD therapy is still underestimated, yet it plays a crucial role, not only in performing recommended relaxation exercises. The authors’ extensive experience and literature data indicate the multifaceted benefits of patient involvement in therapy, following prior, thorough education.

Modern literature emphasizes the importance of the various methods used to treat the disorder, but too little attention is paid to the implementation of therapeutic recommendations in the course of the long and complex treatment of TMD [1,5,8,12,19,21,22,23,24,25,26].

## 5. Conclusions

The authors did not find any articles that precisely describe the verification of the application of general therapeutic recommendations intended for patients. The unfavorable results of our own studies indicate that the provision of recommendations requires a longer time and a detailed explanation of why the implementation of recommendations is so important in the course of the TMD treatment process. Another beneficial implication may be the need to educate primary care physicians about the principles of TMD prevention. We emphasize the necessity to devote more time and provide detailed explanations to patients on why adherence is critical for treatment success. This recommendation constitutes an important implication of our findings.

## Figures and Tables

**Table 1:** Analysis of frequency of adherence to recommendations.

Recommendations	*N*	%
Application of physiotherapy treatments in a series of 10 to 14 treatments at small intervals	48	60.0%
Stress management/education of ways to cope with stress	46	57.5%
Sleep hygiene	46	57.5%
Maintaining dental arch discomfort throughout the day	46	57.5%
Performing 15 repetitions of relaxation exercises daily throughout the day	44	55.0%
Using an orthopaedic pillow while sleeping	43	53.8%
Required duration of occlusal splint use	38	47.5%
Hot compresses on the chewing muscles	37	46.3%
Taking recommended supplements	37	46.3%
Mental control of the jaw position/fighting against pathological habits of teeth clenching	37	46.3%

**Table 2:** Descriptive statistics with the Shapiro-Wilk test.

	*M*	*Mdn*	*SD*	*Sk.*	*Kurt.*	*Min.*	*Max.*	*W*	*p*
Adherence rate	5.28	5.00	2.15	0.08	−0.85	1	10	0.96	0.011

## Data Availability

The original contributions presented in the study are included in the article, further inquiries can be directed to the corresponding authors.

## References

[1] Wright E., Klasser G. (2020). Manual of Temporomandibular Disorders.

[2] Schiffman E., Ohrbach R., Truelove E., Look J., Anderson G., Goulet J.P., Dworkin S.F. (2014). Diagnostic criteria for temporomandibular disorders (DC/TMD) for clinical and research applications: Recommendations of the International RDC/TMD Consortium Network and Orofacial Pain Special Interest Group. J. Oral Facial Pain Headache.

[3] Fillingim R.B., Ohrbach R., Greenspan J.D., Knott C., Diatchenko L., Dubner R., Maixner W. (2013). Psychological factors associated with development of TMD: The OPPERA prospective cohort study. J. Pain.

[4] Fale H., Hnamte L., Deolia S., Pasad S., Kohale S., Sen S. (2018). Association between parafunctional habit and sign and symptoms of temporomandibular dysfunction. J. Dent. Res. Rev..

[5] Tatli U., Benlidayi M.E., Ekren O., Salimov F. (2017). Comparison of the effectiveness of three different treatment methods for temporomandibular joint disc displacement without reduction. Int. J. Oral Maxillofac. Surg..

[6] Wänman A., Ernberg M., List T. (2016). Guidelines in the management of orofacial pain/TMD. An evidence-based approach. Nor. Tann. Tid..

[7] Pesqueira A.A., Zuim P.R., Monteiro D.R., Do Prado Ribeiro P., Garcia A.R. (2010). Relationship between psychological factors and symptoms of TMD in university undergraduate students. Acta Odontol. Latinoam..

[8] Tran C., Ghahreman K., Huppa C., Gallagher J.E. (2022). Management of temporomandibular disorders: A rapid review of systematic reviews and guidelines. Int. J. Oral Maxillofac. Surg..

[9] Slade G.D., Diatchenko L., Bhalang K., Sigurdsson A., Fillingim R.B., Belfer I., Maixner W. (2007). Influence of psychological factors on risk of temporomandibular disorders. J. Dent. Res..

[10] Paço M., Peleteiro B., Duarte J., Pinho T. (2016). The effectiveness of physiotherapy in the management of temporomandibular disorders: A systematic review and meta- analysis. J. Oral Facial Pain Headache.

[11] Song Y.L., Yap A.U. (2018). Outcomes of therapy TMD interventions on oral health related quality of life: A qualitative systematic review. Orofac. Pain.

[12] Ferrillo M., Giudice A., Marotta N., Fortunato F., Di Venere D., Ammendolia A., de Sire A. (2022). Pain management and rehabilitation for central sensitization in temporomandibular disorders: A comprehensive review. Int. J. Mol. Sci..

[13] Butts R., Dunning J., Pavkovich R., Mettille J., Mourad F. (2017). Conservative management of temporomandibular dysfunction: A literature review with implications for clinical practice guidelines (Narrative review part 2). J. Bodyw. Mov. Ther..

[14] De Freitas R.F.C.P., Ferreira M.Â.F., Barbosa G.A.S., Calderon P.S. (2013). Counselling and self-management therapies for temporomandibular disorders: A systematic review. J. Oral Rehabil..

[15] Nicolakis P., Erdogmus B., Kopf A., Nicolakis M., Piehslinger E., Fialka- Moser V. (2002). Effectiveness of exercise therapy in patients with myofascial pain dysfunction syndrome. J. Oral Rehabil..

[16] Fernández-de-las-Peñas C., Von Piekartz H. (2020). Clinical Reasoning for the Examination and Physical Therapy Treatment of Temporomandibular Disorders (TMD): A Narrative Literature Review. J. Clin. Med..

[17] Ho R. (2017). Understanding Statistics for the Social Sciences with IBM SPSS.

[18] Gauer R.L., Semidey M.J. (2015). Diagnosis and treatment of temporomandibular disorders. Am. Fam. Physician.

[19] Pihut M., Pihut M., Ferendiuk E., Szewczyk M., Kasprzyk K., Wieckiewicz M. (2016). The efficiency of botulinum toxin type A for the treatment of masseter muscle pain in patients with temporomandibular joint dysfunction and tension-type headache. J. Headache Pain.

[20] Pihut M., Szuta M., Ferendiuk E., Zeńczak-Więckiewicz D. (2014). Evaluation of pain regression in patients with temporomandibular dysfunction treated by intra- articular platelet-rich plasma injections: A preliminary report. BioMed Res. Int..

[21] Martins W.R., Blasczyk J.C., de Oliveira M.A.F., Gonçalves K.F.L., Bonini- Rocha A.C., Dugailly P.M., de Oliveira R.J. (2016). Efficacy of musculoskeletal manual approach in the treatment of temporomandibular joint disorder: A systematic review with meta-analysis. Man. Ther..

[22] Doeuk C., Hersant B., Bosc R., Lange F., SidAhmed-Mezi M., Bouhassira J., Meningaud J.P. (2015). Current indications for low level laser treatment in maxillofacial surgery: A review. Br. J. Oral Maxillofac. Surg..

[23] Li D.T.S., Leung Y.Y. (2021). Temporomandibular disorders: Current concepts and controversies in diagnosis and management. Diagnostics.

[24] de Barreto Aranha R.L., De Abreu M.H.N.G., Serra-Negra J.M., Martins R.C. (2018). Current evidence about relationships among prosthodontic planning and temporomandibular disorders and/or bruxism. J. Evid. Based Dent. Pract..

[25] Gil-Martínez A., Paris-Alemany A., López-de-Uralde-Villanueva I., La Touche R. (2018). Management of pain in patients with temporomandibular disorder (TMD): Challenges and solutions. J. Pain Res..

[26] Lile I.E., Hajaj T., Veja I., Hosszu T., Vaida L.L., Todor L., Stana O., Popovici R.-A., Marian D. (2025). Comparative Evaluation of Natural Mouthrinses and Chlorhexidine in Dental Plaque Management: A Pilot Randomized Clinical Trial. Healthcare.

